# Phenotypic and genotypic characterization of macrolide, lincosamide and streptogramin B resistance among clinical isolates of staphylococci in southwest of Iran

**DOI:** 10.1186/s13104-018-3817-4

**Published:** 2018-10-10

**Authors:** Reza Khashei, Yalda Malekzadegan, Hadi Sedigh Ebrahim-Saraie, Zahra Razavi

**Affiliations:** 1 Department of Bacteriology and Virology, School of Medicine, Shiraz University of Medical Sciences, Shiraz, Iran; 2 Student Research Committee, Shiraz University of Medical Sciences, Shiraz, Iran

**Keywords:** Inducible resistance, *Staphylococcus*, Clindamycin, Erythromycin, *erm* genes

## Abstract

**Objective:**

The present study aimed to determine the phenotypic and genotypic profile of macrolide, lincosamide and streptogramin B (MLS_B_) resistance in clinical isolates of staphylococci.

**Results:**

This cross-sectional study was conducted on 164 non-duplicated staphylococci isolates collected during August 2015 to February 2016 from two tertiary care hospitals in Shiraz, southwest of Iran. Of the 164 isolates, 86 erythromycin-resistant isolates consist of 35 *Staphylococcus aureus* and 51 coagulase negative staphylococci (CoNS) were included in the study. Of the 35 *S. aureus*, the prevalence of cMLS (constitutive), iMLS (inducible), and MS phenotypes were found 82.9%, 8.6% and 8.6%, respectively. Among 51 CoNS, the frequencies of cMLS, iMLS, and MS phenotypes were detected 66.7%, 11.8% and 21.6%, respectively. Among *S. aureus* isolates, the predominant genes were *ermC* in 82.9% isolates, followed by *ermA* in 57.1% and *msrA* in 28.6% of isolates. Among CoNS isolates, the most frequent genes were diagnosed *ermC* in 70.6% isolates followed by *msrA* in 68.6% and *ermA* in 11.8% of isolates. In conclusion, regarding the presence of MLS_B_ resistance in our region, diagnosis of this resistance type on a routine basis in staphylococcal clinical isolates is of particular importance.

## Introduction

Staphylococci are amongst the most frequent causes of nosocomial and community-acquired infections worldwide [1, 2]. The emergence of antibiotic resistant *Staphylococcus aureus*, particularly methicillin resistant *Staphylococcus aureus* (MRSA) pose difficulties in treatment of related infections [3]. Macrolide, lincosamide and streptogramin B (MLS_B_) antibiotics are one the available options for treating staphylococcal infections [4]. These antibiotics are used in treatment of a wide range of bacterial infections; however, frequently used as drug of choice to treat staphylococcal skin and soft tissue infections (SSTIs) [5]. Recently, the increasing prevalence of methicillin resistance and other chemotherapeutic agents among staphylococci become a global health concern [6].

Resistance to MLS antibiotics in *S. aureus* and coagulase negative staphylococci (CoNS), can resulted in the target site modification encoded by *erm* genes [7, 8]. The methylation of the 23S rRNA conferred by *erm* genes prevents the binding of antibiotic to its ribosomal target [9]. Other mechanisms are efflux pumps encoded by *msrA* gene which mediated resistance to MS_B_, and the drug modification encoded by *lnu* gene [10, 11].

MLS_B_ phenotype can be expressed into forms of constitutive (cMLS_B_) or inducible (iMLS_B_) [12]. Constitutive resistance is related to *S. aureus* strains which are resistant to both erythromycin and clindamycin [13]. Inducible strains define those bacteria which are actually resistant to erythromycin and clindamycin, but are susceptible to clindamycin by routine susceptibility tests [13]. Constitutive MLS_B_ resistance do not require specific method for routine detection, whereas iMLS_B_ resistance is not recognized using the standard susceptibility methods [14]. Erythromycin is the effective inducer of iMLS_B_ resistance than clindamycin. Owed to this fact, iMLS_B_ resistance can be determined by special disk approximation tests that incorporate erythromycin induction of clindamycin resistance (D-zone effect) [14].

Clindamycin treatment in patients with iMLS_B_ resistance may lead to development of cMLS_B_ resistant strains and subsequently therapeutic failure [12]. Therefore, it is important to identify actual MLS_B_ resistance for establishing appropriate therapy in infected patients. Due to the lack of data on the prevalence and characteristics of MLS_B_ resistant strains in our region, we aimed to determine the resistance rates and predominant resistance mechanisms toward MLS_B_ antibiotics among clinical isolates of staphylococci obtained from Iranian patients.

## Main text

### Methods

#### Study design and identification of isolates

This cross-sectional study was conducted on 164 non-duplicated staphylococci isolates collected from two major teaching hospitals, Nemazee and Faghihi, in Shiraz, southwest of Iran. Staphylococci isolates were recovered from different body sites such as blood, wound, sputum, urine and other clinical specimens between August 2015 and February 2016. Standard microbiological techniques, including colony morphology, Gram stain, catalase test, tube coagulase test, DNase test and growth on mannitol salt agar were used for identification of *S. aureus* and CoNS isolates.

#### Antibiotic susceptibility testing

Antibiotic susceptibility test was performed on Muller Hinton agar (Merck, Germany) using the disk diffusion method according to Clinical and Laboratory Standards Institute (CLSI) guidelines [15]. Methicillin resistance was primary detected based on resistance to cefoxitin (30 μg) disk (Rosco, Denmark) by CLSI recommended disk diffusion method. The isolates which were initially susceptible to clindamycin (2 μg) and resistant to erythromycin (15 μg) were examined for inducible clindamycin resistance using the D-test according to CLSI recommendations [15]. Briefly, erythromycin (15 μg) and clindamycin (2 μg) disks were placed 15–20 mm apart (edge to edge) and then incubated at 35–37 °C for 18 h. Staphylococcal isolates showing resistance to erythromycin but being susceptible to clindamycin and producing a D-shaped zone of inhibition around the clindamycin disk on the side facing the erythromycin disk were considered as iMLS resistance phenotype. Moreover, resistance to both erythromycin and clindamycin was indicated a cMLS resistance phenotype. Staphylococcal isolates showing resistance to erythromycin while being susceptible to clindamycin with no blunting zone were classified as the MS resistance phenotype. *S. aureus* ATCC 25923 was used as a control strain for antibiotic susceptibility testing.

#### DNA extraction and polymerase chain reaction (PCR) assay

Bacterial whole DNAs were extracted from Staphylococcal isolates using the boiling method and used as PCR templates. All *S. aureus* isolates, including methicillin sensitive *Staphylococcus aureus* (MSSA) and MRSA were subsequently tested for the presence of *femA* and *mecA* genes by a set of previously described primers [16, 17]. PCR reaction was performed for detection of erythromycin resistance determinants (*ermA*, *ermB*, *ermC*, and *msrA* genes) [18], and two major virulence factors of *S. aureus* (*tst*-*1*, and *pvl* genes) with specific primers [17, 19]. The reference strains were provided from our colleague previous work and used as positive controls in PCR experiments [20]. PCR amplifications were performed in a DNA Thermal Cycler 5530 (Ependrof master, Germany). PCR products were mixed with 1 µl loading buffer solution and were loaded into the wells of agarose gel (1.5%) carefully and electrophoresed at 75 V for 90 min. The gel was then stained with KBC (Merck, Germany) solution for 15 min and observed under the UV trans-illuminator.

#### Statistical analysis

Data were analyzed using SPSS™ software (IBM corp., USA) version 21.0. The results were presented as descriptive statistics in terms of relative frequency. Chi–square or Fisher’s exact tests were used to estimate any statistical association for quantitative variables. Values were expressed as the mean ± standard deviation (continuous variables) or percentages of the group (categorical variables). Statistical significance was regarded as *P*-values < 0.05.

### Results

Totally, 164 staphylococci clinical isolates consisting of 97 *S. aureus* and 67 CoNS were included in the study. Of 164 isolates of staphylococci, 86 erythromycin-resistant isolates consisting of 35 *S. aureus* and 51 CoNS were examined. Of 86 isolates, 51 (59.3%) and 35 (40.7%) were from male and female patients, respectively with a mean age of 37.1 ± 27.2 years, ranging from 1 month to 109 year old. The most common sites of bacterial isolation were from bloodstream infections (BSIs) with frequencies 40 (46.5%), followed by SSTIs 16 (18.6%), urinary tract infections (UTIs) 13 (15.1%), respiratory tracts infections (RTIs) 12 (14%), eye infections 3 (3.5%), abdominal infections 1 (1.2%), and bone and joint infections 1 (1.2%). The *mecA* gene screening showed that 32 of the tested isolates were MRSA and 44 were methicillin-resistant coagulase-negative staphylococci (MRCNS).

Of 35 *S. aureus*, the overall prevalence of cMLS, iMLS, and MS phenotypes were 29 (82.9%), 3 (8.6%), and 3 (8.6%), respectively. Among 51 CoNS, the overall prevalence of cMLS, iMLS, and MS phenotypes were 34 (66.7%), 6 (11.8%), and 11 (21.6%), respectively. As shown in Table 1, among *S. aureus* isolates the predominant genes were *ermC* in 29 (82.9%) isolates, followed by *ermA* in 20 (57.1%) and *msrA* in 10 (28.6%) isolates. Among CoNS isolates the most prevalent genes were *ermC* in 36 (70.6%) isolates, followed by *msrA* in 35 (68.6%) and *ermA* in 6 (11.8%) isolates. Meanwhile, *ermB* gene was not found among all of the tested isolates. Interestingly, in one of the MSSA isolates with cMLS phenotype any of investigated genes was not found.

**Table 1:** The frequency of phenotypic and genotypic resistance to MLS according to type of isolates

Methicillin-resistance type	Phenotype	*ermA*	*ermC*	*msrA*	*tst*-*1*	*pvl*
MRSA (N = 32)	cMLS (N = 27)	18	25	6	7	1
	iMLS (N = 2)	1	1	0	1	0
	MS (N = 3)	1	1	3	2	0
MSSA (N = 3)	cMLS (N = 2)	0	1	0	1	0
	iMLS (N = 1)	0	1	1	1	0
	MS (N = 0)	–	–	–	–	–
Total (N = 35)		20 (57.1%)	29 (82.9%)	10 (28.6%)	12 (34.3%)	1 (2.9%)
MRCoNS (N = 48)	cMLS (N = 34)	6	31	19	ND	ND
	iMLS (N = 6)	0	5	5		
	MS (N = 8)	0	0	8		
MSCoNS (N = 3)	cMLS (N = 0)	–	–	–	ND	ND
	iMLS (N = 0)	–	–	–		
	MS (N = 3)	0	0	3		
Total (N = 51)		6 (11.8%)	36 (70.6%)	35 (68.6%)	–	–
P value		< 0.001	0.19	< 0.001	–	–

ND, not determined

The details of macrolides resistance phenotypic and genotypic related to the source of infections are presented in Table 2. Moreover, in urinary tract, eye and abdominal originated specimens no *ermA* gene was found. On the other hand, the most common isolation sites of staphylococci with iMLS phenotype were bloodstream, skin and soft tissue, urinary tract, and eye.

**Table 2:** Macrolides resistance phenotypic and genotypic characterization in relation to source of infections

Phenotype	*ermA*	*ermC*	*msrA*	*tst*-*1*	*pvl*	cMLS	iMLS	MS
BSIs (40)	11	29	23	2	0	28	5	7
SSTIs (16)	7	12	10	4	1	11	2	3
UTIs (13)	0	11	8	3	0	11	1	1
RTIs (12)	7	10	2	1	0	11	0	1
EIs (3)	0	2	1	2	0	1	1	1
AIs (1)	0	0	1	0	0	0	0	0
BJIs (1)	1	1	0	0	0	1	0	0
Total	26 (30.2)	65 (75.6)	45 (52.3)	12 (34.3%)^a^	1 (2.9%)^a^	63 (73.3%)	9 (10.5%)	14 (16.3)

BSIs, bloodstream infections; SSTIs, skin and soft tissue infections; UTIs, urinary tract infections; RTIs, respiratory tracts infections; EIs, eye infections; AIs, abdominal infections, BJIs, bone and joint infections

^a^The proportion estimated among 35 erythromycin-resistant *S. aureus*

The prevalence of two virulence factors of *S. aureus*, namely toxic shock syndrome toxin (TSST-1), and Panton–Valentine leukocidin (PVL) in 35 erythromycin-resistant isolates by detection of *tst*-*1* and *pvl* genes were 34.3% and 2.9%, respectively. As shown in Table 2, the main source of toxins producing *S. aureus* was SSTIs. Moreover, the proportion of these toxins in all 97 *S. aureus* isolates were 37.1% (36/97) and 3.1% (3/97) for TSST-1 and PVL, respectively.

The combination patterns of genes responsible for resistance to MLS antibiotics in studied isolates are depicted in Table 3. As be shown, 7 different combinations were detected. The most prevalent pattern in *S. aureus* and CoNS were *ermA*/*ermC* (37.1%) and *ermC*/*msrA* (39.2%), respectively.

**Table 3:** The combination patterns of genes responsible for resistance to MLS antibiotics in studied isolates

Pattern	*S. aureus* (N = 35)	CoNS (N = 51)	Total (N = 86)
*ermA*	3 (8.6)	2 (3.9)	5 (5.8)
*ermC*	8 (22.9)	12 (23.5)	20 (23.3)
*msrA*	2 (5.7)	13 (25.5)	15 (17.4)
*ermA*/*ermC*	13 (37.1)	2 (3.9)	15 (17.4)
*ermC*/*msrA*	4 (11.4)	20 (39.2)	24 (27.9)
*ermA*/*ermC*/*msrA*	4 (11.4)	2 (3.9)	6 (7)
No gene	1 (2.9)	0	1 (1.2)

### Discussion

Multi-resistant strains of staphylococci have been reported to frequently acquire resistance to macrolides and related antibiotics, which can led to difficulties in the treatment of infections [21]. Hence, due to variety in the prevalence of these strains in different geographical areas, constant surveillance can be helpful to control their spread and providing information regarding their current epidemiology. Results of the present study showed new information regarding the occurrence of the MLS resistance in this era of high incidence of MRSA in Iran [22].

In the current study, the overall frequency of iMLS phenotype among *S. aureus* and CoNS were found to be 8.6%, and 11.8%, respectively. Despite the discrepancies, our finding was consistent with previous reports from Iran which had shown low prevalence of iMLS phenotype in staphylococci ranged from 4.1 to 14.9% [11, 23–26]. However, much higher rates (≥ 30%) was also reported by Moosavian et al. and Saffar et al. from Ahvaz and Tehran, respectively [14, 35]. Moreover, the prevalence reported in our study is higher than those reported among staphylococci isolated from Brazil (*S. aureus*, 0%) [12], and Mexico (CoNS, 8%) [27], whereas it is lower than those reported from Japan (*S. aureus*, 91%) [28], Korea (*S. aureus*, 34%; CoNS, 90%) [29], Poland (CoNS, 18.7%) [7], Nepal (*S. aureus*, 11.5%) [30], and India (*S. aureus*, 10.8%) [31].

Resistance to MLS antibiotics in staphylococci is mainly mediated by methyltransase encoded by *erm* genes [32]. The distribution of these genes mostly depends on the bacterial species [7]. In our findings, *ermA* gene was more prevalent in *S. aureus* compared to CoNS (P < 0.001). Previously, similar results have been cited by authors from Iran and other countries [9, 23, 33]. In contrast, we found *msrA* gene was more abundant in CoNS (P < 0.001). In agreement with our findings, this gene is the most frequently reported gene in CoNS isolates exhibiting the MLS resistance phenotypes [33, 34]. We did not find any isolates carrying *ermB* gene. Despite the similar reports indicating the prevalence of *ermB* gene in low rates [23, 35], it has been noted that *ermB* is more characteristic of beta-haemolytic streptococci or staphylococci with animal origin [7, 12]. In our study, in one erythromycin-resistant isolate with cMLS phenotype, none of the investigated genes were found. Previously, such discordance among MLS phenotypes and erythromycin resistance genes due to a mutation in the coding or promoter region of targeted genes was reported [32].

In conclusion, regarding the presence of different types of MLS_B_ resistance in our region, diagnosis of these resistance types on a routine basis in staphylococcal clinical isolates is of particular importance. These results suggest that the empiric use of MLS_B_ antibiotics for staphylococcal infections should be prescribed in a logical manner by our physicians.

## Limitations

Finally, as the main limitations of the present study, the lack of evaluation of genes expression by real time-PCR, and genetic relatedness of erythromycin-resistant isolates by a molecular typing method should be acknowledged.

## Abbreviations

MRSA: methicillin resistant *Staphylococcus aureus*
MLS_B_: macrolide, lincosamide and streptogramin B
SSTIs: skin and soft tissue infections
CoNS: coagulase negative staphylococci
MRCNS: methicillin-resistant coagulase-negative staphylococci
cMLS_B_: constitutive macrolide, lincosamide and streptogramin B resistance
iMLS_B_: inducible macrolide, lincosamide and streptogramin B resistance
CLSI: Clinical and Laboratory Standards Institute
